# Research on the Morphology Reconstruction of Deep Cryogenic Treatment on PtRu/nitrogen-Doped Graphene Composite Carbon Nanofibers

**DOI:** 10.3390/ma15030908

**Published:** 2022-01-25

**Authors:** Shuaishuai Lv, Yangyang Zhu, Xingxing Wang, Yu Zhu, Kaixuan Wang, Hongjun Ni, Ruobo Gu

**Affiliations:** 1 School of Mechanical Engineering, Nantong University, Nantong 226019, China; lvshuaishuai@ntu.edu.cn (S.L.); wangxx@ntu.edu.cn (X.W.); zhu.y@ntu.edu.cn (Y.Z.); 1910310003@stmail.ntu.edu.cn (K.W.); 2 Nantong Institute of Technology, Nantong 226019, China; 20200212@ntit.edu.cn; 3 Nantong Vocational College of Science & Technology, Nantong 226019, China; a15052003445@163.com

**Keywords:** cryogenic treatment, composite carbon, morphology reconstruction, electrospinning

## Abstract

To improve the performance of PtRu/nitrogen-doped graphene composite carbon nanofibers, the composite carbon nanofibers were thermally compensated by deep cryogenic treatment (DCT), which realized the morphology reconstruction of composite carbon nanofibers. The effects of different DCT times were compared: 12 h, 18 h, and 24 h. The morphology reconstruction mechanism was explored by combining the change of inner chain structure and material group. The results showed that the fibers treated for 12 h had better physical and chemical properties, where the diameter is evenly distributed between 500 and 800 nm. Combined with Fourier infrared analysis, the longer the cryogenic time, the more easily the water vapor and nitrogen enter polymerization reaction, causing changes of chain structure and degradation performance. With great performance of carbonization and group transformation, the PtRu/nitrogen-doped graphene composite carbon nanofibers can be used as an efficient direct alcohol fuel cell catalyst and promote its commercialization.

## 1. Introduction

Electrospinning is a new controllable preparation technology of nanofibers with strong operability and reproducibility [1]. The fibers prepared by this technology have the advantages of ultra-fine diameter, large specific surface area, and high porosity, which are widely applied in filtration, optoelectronics, energy, biomedicine, and other fields [2]. Woo et al. [3] fabricated the conductive nanofiber network on a flexible substrate via electrospinning, and the overlap between nanofibers was removed by heat treatment. Bhullar et al. [4] prepared TiO_2_ nanofibers based on electrospinning technique, and it has variable diameters (120–450 nm). Generally, the essence of electrospinning fiber is the layer-by-layer superposition of two-dimensional space, causing particles to agglomerate in the fiber when nanoparticles are combined with nanofibers [5]. To solve the above problems, except for changing electrospinning materials and spinning parameters, the post-treatment can be further on carried out to improve the properties of electrospun fiber [6].

Deep cryogenic treatment (DCT) is a special heat treatment method, which generally refers to the cold treatment of raw materials in the environment of −190 to −230 °C. It can improve the microstructure and properties of materials [7]. Mavi et al. [8] investigated the effects of aging and DCT on the hardness and wear behavior of Al7075 alloy, and the test results showed that the applied DCT had positively affected the wear behavior. Singh et al. [9] focused on the effect of DCT on a rotavator blade material of boron steel, which improved by 60% and 260.73% in the abrasive wear resistance and microhardness by DCT. Due to the elimination of residual austenite and initiation of nucleation sites, many very fine carbide particles are precipitated in DCT [10], which can improve the hardness, wear resistance, and other mechanical properties of metal materials [11]. Ozden et al. [12] evaluated the effect of DCT on the microstructure and mechanical properties, which illustrated that applied DCT before the tempering can homogeneously distribute fine carbides. However, the further exploration should be developed to analyze the effect of DCT on the properties of polymer materials [13].

Graphene is a new single-layer carbon atom material with large specific surface area as well as excellent thermal conductivity and mechanical properties [14]. It has good application prospects in various fields [15,16]. With the deepening of research, researchers observed that both sides of graphene can support precious metal substances, so graphene is gradually used as the support material of electrocatalyst [17,18]. Antonini [19] analyzed the effect of a graphene nanolayer on the activity of a catalyst by comparing the carrier carbon black, carbon nanotube, and graphene nanolayer, which can replace traditional carbon black as a catalyst support material for direct alcohol fuel cells. Xiao et al. [20] developed a novel ethanol electrooxidation catalyst based on Pd nanoparticles supported by hydrogenated graphene. The test results suggested that graphene with a high hydrogenation degree can promote the formation and improve the catalytic performance of the catalyst.

In the electrospinning process, the combination of nanoparticles and nanofibers makes it easy to agglomerate the particles in the fibers. If the pre-oxidation is insufficient, the cavity will appear with poor performance. On the contrary, if the pre-oxidation is excessive, it will affect the rearrangement of the structure and carbonization. With the appropriate cryogenic time, the hydrogen and oxygen elements in the organic matter can be removed correctly, which can convert the nitrile group in polyacrylonitrile (PAN) into other groups under the condition of hypoxia or oxygen deficiency.

To explore a more efficient direct alcohol fuel cell catalyst and promote its commercialization, the PtRu/nitrogen-doped graphene (PtRu/NG) composite carbon nanofibers were prepared by electrospinning and heat treatment with the precursors of H_2_PtCl_6_·6H_2_O and RuCl_3_, where the polyacrylonitrile and nitrogen-doped graphene are the carbon sources. By changing the cryogenic time, the effects of cryogenic time were investigated on the microstructure of PtRu/NG composite carbon nanomaterials.

## 2. Materials and Methods

### 2.1. Materials

**Figure 1:**
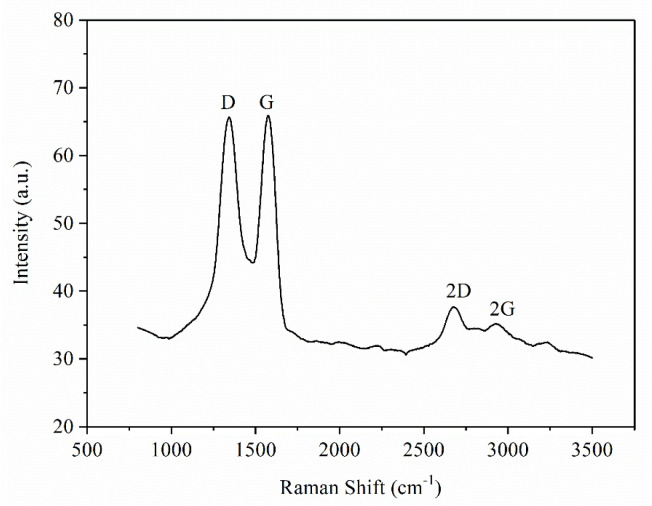
Raman spectrogram of NG.

In the preparation of platinum ruthenium/nitrogen doped graphene composite carbon nanofibers, platinum ruthenium precursors are chloro-platinic acid (H_2_PtCl_6_·6H_2_O, analytical reagent, Shanghai Aladdin Biochemical Technology Co., Ltd., Shanghai, China) and ruthenium trichloride (RuCl_3_·3H_2_O, Beijing Bailing Wei Technology Co., Ltd., Beijing, China); nitrogen sources are polyacrylonitrile (PAN, 150,000, Beijing Bailing Wei Technology Co., Ltd., Beijing, China) and nitrogen-doped graphene (NG, analytical reagent, Shanghai Aladdin Biochemical Technology Co., Ltd., Shanghai, China); solvent is N,N-dimethylformamide (DMF, analytical reagent, Shanghai Aladdin Biochemical Technology Co., Ltd., Shanghai, China). Specially, the Raman spectrogram of NG is shown in Figure 1.

### 2.2. Methods

To analyze the effects of cryogenic time, the methods include two steps: preparation and DCT of PtRu/NG composite carbon nanomaterials.

#### 2.2.1. Preparation of PtRu/NG Composite Carbon Nanofibers

(1) Preparation of precursor solution

After NG of 40 mg is added to DMF of 9 g, it is ultrasonically dispersed for 30 min until forming a uniform mixture. After 0.7 g PAN is added to the solution, it is stirred with a magnetic stirrer at 60 °C for 1 h. RuCl_3_·3H_2_O of 0.0470 g and H_2_PtCl_6_·6H_2_O of 0.35 g (with atomic ratio Pt:Ru = 2:1) are respectively added to the mixed solution and stirred with a magnetic stirrer at the constant temperature 60 °C for 2 h. Eventually, the solute is completely dissolved to thick black solution, namely PtRu precursor solution.

(2) Preparation of fibrous membrane

In the electrostatic spinning of precursor solution, the voltage is 18 kV, the advance speed is 0.002 mm/s, the temperature is 30 °C, and the humidity is 37.5% RH. After 7 h of spinning, a PtRu/NG composite carbon nanofiber is obtained.

Subsequently, the pre-oxidation and carbonization of fibrous membrane are carried out. The process conditions are as follows. Pre-oxidation refers to placing in the air at 250 °C for 2 h. Carbonization means that the pre-oxidized fibrous membrane is kept in argon at 700 °C for 3 h. The untreated, pre-oxidized, and carbonized fibrous membranes are labeled as samples A1, A2, and A3, respectively.

#### 2.2.2. DCT of PtRu/NG Composite Carbon Nanofibers

Through the SLX-30 cryogenic tank (Beijing Zhongkeyuqi Technology Co., Ltd., Beijing, China), the carbonized platinum ruthenium/nitrogen-doped graphene composite carbon nanofibers are subjected to DCT for 12 h, 18 h, and 24 h, respectively. The carbonized fibrous membrane is sealed and placed in a cryogenic tank at the cryogenic temperature of −190 °C for 12 h, 18 h, and 24 h, respectively. When the temperature returned to room temperature, the fibrous membrane is taken out and marked as samples A4, A5, and A6, respectively.

### 2.3. Performance Characterization

An American Thermo DXR Raman spectrometer (Thermo Fisher Scientific(China) Co., Ltd., Shanghai, China) is used to observe the existing form of NG. A Hitachi S-3400N field emission scanning electron microscope (SEM) (Carl Zeiss AG Co., Ltd., Oberkochen, Germany) is used to observe the morphology of platinum ruthenium/nitrogen-doped graphene composite carbon nanofibers in electrostatic spinning before and after DCT. A Brucker D8 GADDS X-ray powder diffractometer (Rigaku Co., Ltd., Tokyo, Japan) is used to characterize the crystal structure, with a scanning range (2θ) of 5°–95° and scanning speed of 7°/min. The Brucker Vertex 70 Fourier transform infrared spectrometer (Bruker(Beijing) Scientific Technology Co., Ltd., Beijing, China) is used to analyze the changes in the molecular structure of the fibrous membrane before and after DCT.

## 3. Results and Discussion

### 3.1. Analysis of Existing Form of NG

**Figure 2:**
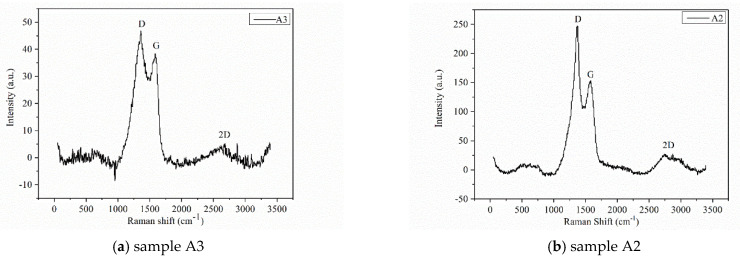
Raman spectrogram of samples A2 and A3.

On the Raman spectrogram of graphene, there are mainly three peaks, namely the D peak, G peak, and 2D peak [21]. The D peak is located at a wave number of 1350 cm^−1^, and its strength is usually used to measure the disorder degree of the material structure. The G peak is located at a wave number of 1580 cm^−1^, which can reflect the graphene membrane number. When the number of layers increases, the G peak moves to the left [22]. In addition, sp^2^ amorphous carbon or diamond-like carbon will cause the G peak to shift to the right [23]. The 2D peak is located at a wave number of 2680 cm^−1^, and its movement and shape are closely related to the graphene membrane number [24]. According to Figure 2a, the D peak, G peak, and 2D peak are relatively obvious. The I_D_/I_G_ = 1.6 and I_G_/I_2D_ > 1.0 indicate the defect density of graphene. The greater the ratio, the greater the degree of defects. In addition, I_D_/I_G_ indicates that graphene exists in multiple layers. Relatively, the Raman spectrogram of sample A2 is obtained, as shown in Figure 2b. When the I_D_/I_G_ = 1.6 and I_G_/I_2D_ = 5 > 1.0, the graphene exists in multiple layers in the A2 sample.

As shown in Figure 1 and Figure 2, there are obvious graphene characteristic peaks of G, D, and 2D. According to the proportion of corresponding peaks, both are multilayer graphene. However, the D peak intensity of the carbonized fiber is weakened, indicating that its disorder degree is improved to obtain a more stable structure and properties.

**Figure 3:**
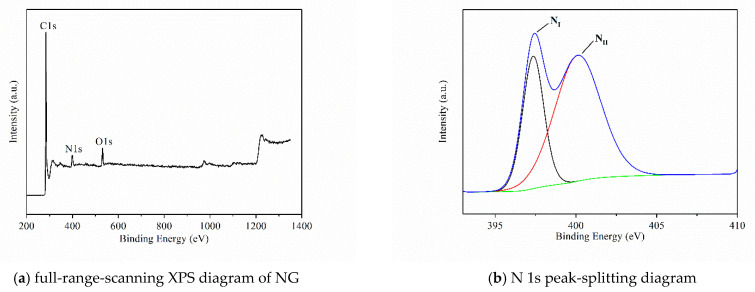
Analysis of morphology for PtRu/NG composite carbon nanofiber.

To investigate the type of nitrogen doping in NG, the surface composition of raw material is analyzed by XPS. The quantitative analysis result of NG is shown in Table 1. The full-range-scanning XPS diagram of NG and N 1s peak-splitting diagram are shown in Figure 3. The XPS spectra were fitted by peaks. It was found that there were two main forms of N 1s, namely pyridine-N (397.2 ev) and pyrrole-N (400.1 ev), with atomic ratios of 52.09% and 47.91%, respectively. Therefore, N in the raw material of NG used for preparing catalyst is mainly the pyridine type, which has good catalytic oxidation activity.

**Figure 4:**
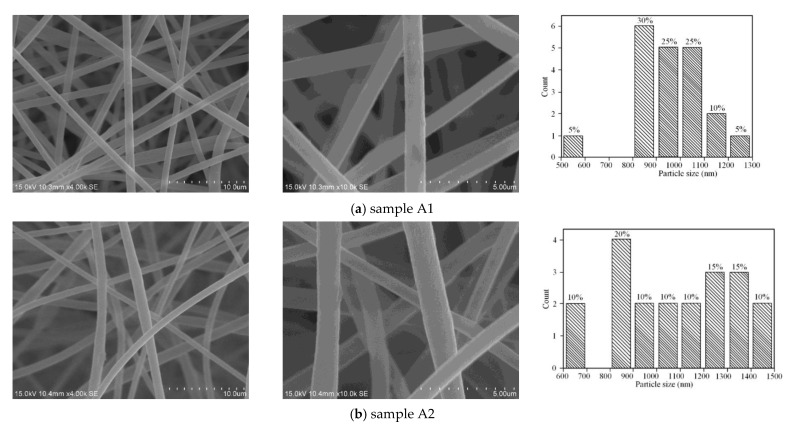

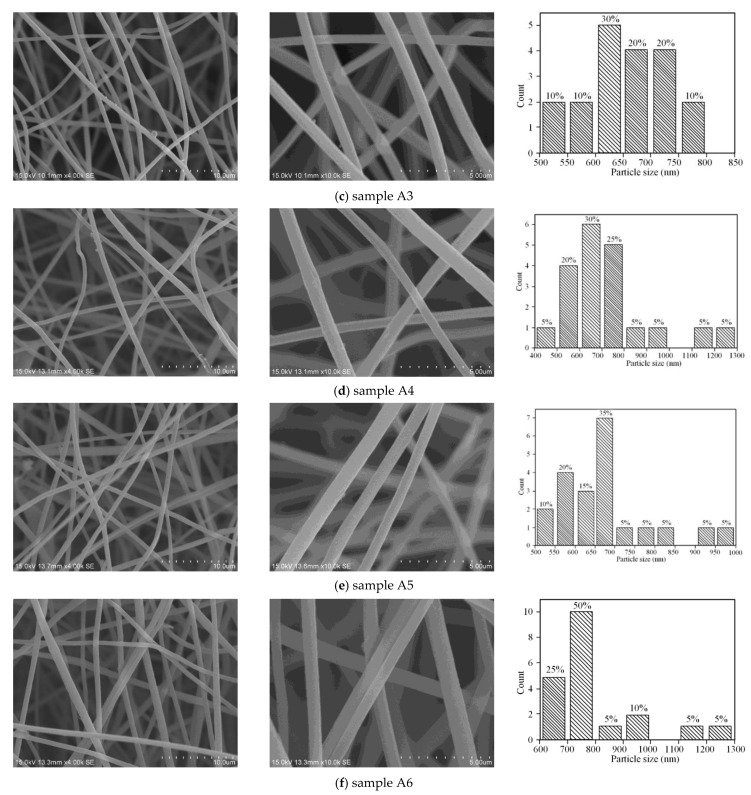
SEM picture of samples A1–A6.

Figure 4 shows the untreated PtRu/NG composite carbon nanofiber membrane SEM with different magnifications, which include 4.0 K and 10.0 K for six samples. Through analysis and comparison, the filament diameter of the untreated fibrous membrane is evenly distributed between 800 and 1100 nm. The diameter of the fibrous membrane after pre-oxidation increases, and the distribution is uneven. This is because the fibrous membrane is under tension during the peroxidation process. The filament diameter of the carbonized fibrous membrane is reduced and the distribution is uniform between 600 and 750 nm, with metal substances attached to the surface of the fiber. The carbonized fibrous membrane forms a carbon fiber after removing a large amount of non-carbon elements. After 12 h of DCT, the diameter of the fibrous membrane is evenly distributed between 500 and 800 nm. After 18 h of DCT, the filament diameter of the fibrous membrane is evenly distributed between 550 and 700 nm. After 24 h of DCT, the filament diameter of the fibrous membrane increased to between 600 and 800 nm, and large grooves appeared. Therefore, it can be obtained that DCT will not damage the internal structure of the fiber, which may be due to material volume shrinkage and carbon precipitation [25].

Generally, the internal molecules recombine through the secondary bonds of intramolecular hydrogen bonds during DCT. It can enhance the intermolecular force of the fiber, improve the molecular binding energy, and increase the tightness of the molecular chain, which can improve the crystallinity of the material. In addition, the macromolecular structure inside the fiber is more compact and regular.

### 3.2. Analysis of Group Transformation for PtRu/NG Composite Carbon Nanofiber

**Figure 5:**
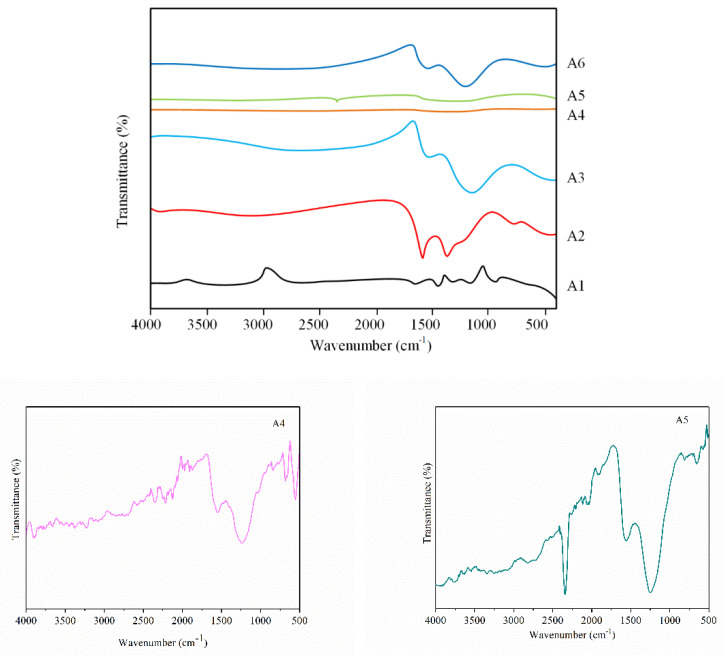
Infrared spectrogram of samples A1–A6.

A Fourier infrared test is used to compare and analyze the internal chain structure and internal group changes of six samples, as shown in Figure 5. Generally, after adding the PAN into the precursor, the molecular chain of droplets undergoes a series of complex reactions such as cyclization, crosslinking, dehydrogenation, and oxidation due to tension. The intramolecular cyclization, intermolecular cyclization, and oxidative decomposition mainly appeared at 215 °C, 300 °C, and 380 °C, respectively. In addition, the PAN is transformed into a pyridine ring trapezoidal structure with high thermal stability, which can improve the heat resistance and conductivity. Therefore, pre-oxidation is the key to determine the structure and properties of carbon fibers, which is also one of the key steps in the preparation of fiber membrane.

By comparing the infrared spectrograms of samples A1 and A2, it can be found that various chemical changes occur during the pre-oxidation of that fibrous membrane, including cyclization reaction, deoxidation reaction, and oxidation reaction. The structure of PAN is transformed into a stable trapezoidal six-member ring structure, -COOH. Extensional vibration CH3 is converted to other groups. Oxygen is directly combined with the pre-oxidation wire structure to form an epoxy group. However, there are still C≡N groups at wave numbers of 2100–2400 cm^−1^.

On the other hand, the carbonization is mainly to remove hydrogen and oxygen elements from organic matter, which can convert nitrile groups in PAN into other groups under an anoxic or oxygen-deficient state. PAN is transformed into a carbon fiber with a two-dimensional disordered graphite structure after carbonization. It can further cyclize, dehydrate, dehydrogenate, and decompose the unreacted PAN during pre-oxidation.

According to the infrared spectrogram of sample A3, it can be found that there are three absorption bands near the wave numbers of 1600. According to the infrared spectrogram of sample A3, it can be found that there are three absorption bands near the waves of 1600 cm^−1^, 1500 cm^−1^, and 1450 cm^−1^, namely the framework of aromatic ring C=C. At the same time, C≡N basically disappears after carbonization and is transformed into other groups. Therefore, pre-oxidation and carbonization make PAN into carbon fiber, 1500 and 1450 cm^−1^, namely the framework of aromatic ring C=C. At the same time, C≡N basically disappears after carbonization and is transformed into other groups. Therefore, pre-oxidation and carbonization make PAN into carbon fiber.

In the spectrogram of sample A4, the 1600 cm^−1^ band splits into 1600 and 1580 cm^−1^, which causes the aromatic ring to conjugate with other unsaturated systems. However, there are C≡N groups in the spectrograms of samples A5 and A6.

It can be seen that the longer the cryogenic time is, the less favorable it is for PAN to form carbon fiber. Considering that DCT mainly adopts the principle of liquid nitrogen vaporization, a trace amount of water vapor [26] and nitrogen will inevitably enter in the cryogenic modification process, which may cause chain polymerization and other reactions, which will lead to the reappearance of C≡N groups.

### 3.3. Morphology Reconstruction of PtRu/NG Composite Carbon Nanofiber

In the preparation of platinum ruthenium/nitrogen-doped graphene composite carbon nanofibers, RuCl_3_·3H_2_O and H_2_PtCl_6_·6H_2_O are used as platinum ruthenium precursors. By changing the duration of DCT, platinum ruthenium/nitrogen-doped graphene composite carbon nanofibers with different physical and chemical properties are obtained. In the process of DCT, the inside of the molecule is reorganized. Through the recombination of intramolecular hydrogen bonds and secondary bonds, the force between the fiber molecules, molecular interaction, and the tightness of molecular chains are improved, so that the material crystallinity is improved, and the macromolecular structure inside the fiber is more compact and regular. However, long-term DCT will cause the entry of water molecules and nitrogen elements, which will affect the degree of fiber orientation and cause intra and intermolecular hydrogen bond destruction and fiber defects.

**Figure 6:**
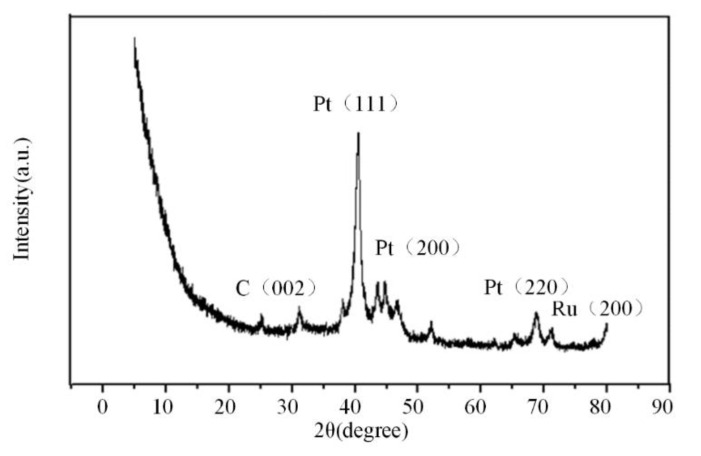
XRD diagram of PtRu/NG catalyst membrane.

In addition, the carbonized PtRu/NG catalyst membrane is tested by XRD, and the results are shown in Figure 6. The corresponding diffraction peaks are detected at 2θ of 40.246°, 46.814°, and 68.361°, respectively. Compared with the standard card of Pt, the three peaks are Pt (111), Pt (200), and Pt (220). Therefore, the detected Pt is a face-centered cube (FCC) structure. The diffraction peaks of the catalyst are negatively shifted, which indicates that Ru diffuses into the lattice of Pt to form PtRu alloy. In addition, the C (002) appears at 24.846°, which also verifies the existence of graphene.

## 4. Conclusions

(1) Through the combination of electrostatic spinning, carbonization, and DCT, the morphology reconstruction of platinum ruthenium/nitrogen-doped graphene composite carbon nanofibers is carried out, thus realizing the application of cryogenic modification fibers;
(2) DCT can effectively improve molecular interaction and material crystallinity, providing the possibility for the preparation of nanoscale materials;
(3) Cryogenic time should not be too long under the condition of treating the fiber at the same cryogenic temperature. The longer the cryogenic time, the greater the environmental impact, and the more likely the fiber is to produce defects.
(4) DCT has a significant effect on the nanofiber, and there have not been many studies on the mechanism and application of DCT in nanofiber materials. Therefore, an in depth exploration of the mechanism is of great importance in order to obtain universal laws and applications.

The research results obtained can provide a certain reference value for the application of PtRu/NG composite carbon nanofiber in fuel cell and other fields. In addition, two aspects need to be further developed and studied in the future. Specifically, it includes the effects of cryogenic treatment at different temperatures on the catalytic membrane and different heat treatment methods on the electrical properties of catalytic membranes.

## Figures and Tables

**Table 1:** Quantitative analysis results of NG.

Sample	C 1s		O 1s		N 1s	
	E_B_/eV	Atomic Fraction/%	E_B_/eV	Atomic Fraction/%	E_B_/eV	Atomic Fraction/%
NG	284.28	88.30	531.21	5.18	399.18	6.52

## Data Availability

The data presented in this study are available on request from the corresponding author.
